# Evaluation of Risk Factors Associated With Herds With an Increased Duration of Bovine Tuberculosis Breakdowns in Castilla y Leon, Spain (2010–2017)

**DOI:** 10.3389/fvets.2020.545328

**Published:** 2020-09-25

**Authors:** Pilar Pozo, Beatriz Romero, Javier Bezos, Anna Grau, Jesus Nacar, Jose Luis Saez, Olga Minguez, Julio Alvarez

**Affiliations:** ^1^VISAVET Health Surveillance Centre, Universidad Complutense de Madrid, Madrid, Spain; ^2^MAEVA SERVET, S.L., Madrid, Spain; ^3^Departamento de Sanidad Animal, Facultad de Veterinaria, Universidad Complutense de Madrid, Madrid, Spain; ^4^Dirección General de Producción Agropecuaria e Infraestructuras Agrarias, Consejería de Agricultura y Ganadería de la Junta de Castilla y León, Valladolid, Spain; ^5^Subdirección General de Sanidad e Higiene Animal y Trazabilidad, Dirección General de la Producción Agraria, Ministerio de Agricultura, Pesca y Alimentación, Madrid, Spain

**Keywords:** bovine tuberculosis, *Mycobacterium bovis*, chronic breakdowns, risk factors, cattle, case-control study, survival analysis

## Abstract

The persistence of bovine tuberculosis (bTB) in certain cattle herds is a major concern in countries pursuing disease eradication worldwide. The chronic nature of the disease, the lack of performance of diagnostic tools, and the presence of wildlife reservoirs may lead infected herds to require longer periods to achieve the officially tuberculosis-free (OTF) status. Here, we evaluated the impact of farm and breakdown characteristics on the probability of disease persistence in infected farms in Castilla y Leon, a bTB-endemic region of Spain, using survival and logistic regression models. Data from bTB breakdowns occurring in 3,550 bTB-positive herds detected in 2010–2017 were analyzed. A multivariable Cox proportional hazards model was fitted using time to recover OTF status as the response variable, and a multivariable logistic regression model using the chronic status (yes/no) for herds experiencing particularly long breakdowns as the outcome variable was also used. Both analyses revealed that county-level bTB herd prevalence, herd size, number of incoming animals in the previous 3 years, number of skin test reactors in the disclosing test, and number of days between the disclosing and follow-up tests were associated with increased breakdown duration. Production type was not consistently associated with chronic infection, suggesting that once infected, it is not a significant predictor of outbreak duration beyond the initial stages of the breakdown. Province-level location and number of animals that are bacteriology-positive also affected significantly the expected herd breakdown duration, but their effect became less significant over time. Risk factors identified in this study may help to identify herds more prone to suffer chronic bTB infection that may require additional control measures early on in a breakdown.

## Introduction

Bovine tuberculosis (bTB) is a zoonotic disease affecting cattle caused by members of the *Mycobacterium tuberculosis* complex, mainly *Mycobacterium bovis* and *Mycobacterium caprae*, which has a major impact in the economy of affected countries due to its effect on trade. Despite being eradicated in several countries (1, 2), bTB is still a challenge in many others (3). In Spain, bTB is still present in several regions, and although the implementation of a national eradication program has led to a decrease in the herd prevalence in the last decades, current levels are still similar to those recorded 17 years ago (2.2% in 2002 and 2.3% in 2018) (4).

Accurate diagnosis of bTB in live animals is often difficult, and several factors that influence test performance and can hence lead to possible diagnostic failures have been identified. These include the intrinsic limited sensitivity of currently available tests, the choice of the diagnostic cutoff, the test procedure, the disease stage of infected animals, the possible desensitization to the test in the case of the skin test, the existence of host or pathogen genetic variations, the occurrence of cross-reactions, and the effect of concurrent infections (5–8). Other factors that impede disease eradication are survival and persistence of *M. bovis* in the environment and the aggregation at communal water and food sources that can promote closer contacts between cattle and may increase the likelihood of contact with infected wildlife reservoirs (9).

An extensively assessed feature of bTB is its persistence in certain herds, in terms of either herd recurrence or prolonged periods of restriction (10, 11). These may imply reinfection, which could be attributed to a local source (such as a contaminated environment, infected wildlife, or farm-to-farm contacts with infected neighboring herds) or to the entry of undetected infected animals and/or ongoing transmission due to residual (persistent but undetected) infection (12). Infection persistence in a herd may indicate a test failure, thereby allowing false-negative infected animals not only to remain in the population but to potentially act as an ongoing source of infection to other herds and wildlife (13, 14).

A large proportion of bTB-positive herds in Spain is located in areas where conditions that favor disease persistence are relatively common, such as the presence of potential wildlife reservoirs (mostly red deer and wild boar) and the predominance of extensively managed bullfighting and beef herds (15, 16). The Castilla y Leon autonomous community, which holds 20% of Spain's total cattle population, is classified as a high-prevalence region (>1%) in the country (4) and has areas that meet the requirements described above to hamper the progress of the eradication program. Out of 3,550 positive herds detected during the period 2010–2017, bTB infection was confirmed through bacteriological culture in 41% of them. Of these, >70% tested bTB positive for 2 or more years, showing that a major part of the bTB burden detected in the region is concentrated on a subset of herds. A study performed on the cattle movement network demonstrated that infected herds in this region were clustered in space but not in the movement network, suggesting that factors other than movements may be related to disease introduction and maintenance in at least a proportion of positive farms (17).

Although several studies have been conducted on herd-level risk factors for bTB in Spain (18, 19), to date, factors contributing to these persistently infected herds experiencing particularly long outbreaks have not been clarified. To improve our understanding on why certain herds have a higher risk of experiencing prolonged bTB breakdowns, we examined the impact of farm characteristics (such as production type, herd size, and animal trade flow) and bTB breakdown-specific variables (such as results in the initial bTB tests and bTB prevalence in the region) on the duration of bTB breakdowns during the 2010–2017 period in Castilla y Leon. This study may provide useful information on the characteristics that influence the persistence of bTB in highly prevalent areas and help in the design of targeted strategies to manage bTB-positive herds in Spain.

## Materials and Methods

### Bovine Tuberculosis Eradication Program in Castilla y Leon

In accordance with EU Directive 64/432/EEC (20), the Spanish bTB eradication program is based on test and slaughter surveillance and live animal testing using in Castilla y Leon the single intradermal test (SIT) in all herds (except certain fattening herds in which compulsory testing may not be required if these only move cattle to the slaughterhouse) plus the interferon-gamma (IFN-γ) assay as a complementary test in infected herds to maximize the diagnostic sensitivity. Briefly, all animals above 6 weeks old in tested herds are subjected to routine SIT herd tests by intradermal inoculation of 0.1 ml of bovine purified protein derivative (PPD) (Cz Veterinaria, Porriño, Spain) in the anterior neck area with a frequency dependent on the bTB prevalence in their local area (ranging from 1–2 every year); after 72 h, animals with a >2-mm increase in skinfold thickness and/or presence of necrosis, edema, exudation, or inflammation of lymph nodes peripheral to the inoculation site are considered reactors (severe interpretation), culled within the following 15 days and subjected to postmortem analysis. Subsequently, OTF status is either suspended or withdrawn, and movement restrictions are applied. Farms confirmed as infected (≥1 positive animal in bacteriological culture and/or epidemiological evidence such as forming a single epidemiological unit sharing facilities with other herds where bTB has been confirmed) are then subjected to follow-up tests using the SIT and IFN-γ tests that must be conducted within the following 2–6 months until they recover the OTF status (two consecutive negative herd tests separated by at least 60 days; Supplementary Figure 1). During the study period, two versions of the IFN-γ assay (using bovine and avian PPDs, CZ Veterinaria) were authorized for application in the region according to the Spanish National Eradication Program: during 2010–2016, the Bovigam IFN-γ test (cutoff value of 0.05, Bovigam®, Thermo Fisher Scientific, Waltham, MA, USA), and in 2016–2017, the IDvet IFN-γ test (manufacturer-recommended cutoff value of 35, ID Screen® Ruminant IFN-γ, IDvet, Grabels, France) (21, 22). Additionally, pre-movement tests within 30 days prior to the animal movement are routinely performed in all herds (4). When bTB is suspected in a herd (i.e., when reactors are found), the official veterinary services conduct an epidemiological investigation in which they collect information about the herds where the reactor(s) resided and its movements in the 2 years prior to bTB confirmation, the neighboring herds, and other possible sources of infection.

### Data Sources

The primary population was comprised of all cattle herds in Castilla y Leon subjected to bTB testing during 2010–2017. Information on herd characteristics, cattle movements, and bTB status of farms was collected through the SITRAN Information System (23). Demographic information available for each herd included its unique identification number, herd production type (beef, fattening, bullfighting, breeding heifers, dairy or mixed—beef, and dairy), and number of animals present at the beginning of each year. Considering the similar management in dairy–mixed herds and the limited number of fattening units compared to beef herds (see *Results*), these were grouped into two categories for simplicity (dairy/mixed and beef/fattening, respectively). Additionally, data about the number of movements (contacts) and animals received by each farm during 2007–2016 were also available. Finally, information on the date and type of bTB test (routine testing, pre-movement test, or follow-up tests in bTB-positive farms), number of animals tested, and number of reactors found in the SIT and/or IFN-γ assay for herd tests performed in the frame of the bTB eradication program during 2007–2018 was collected. For herds subjected to whole-herd depopulation, information on the date of depopulation was also obtained. The total number of cattle older than 6 weeks tested per herd in routine and follow-up tests was used to calculate herd size.

### Study Definitions and Duration of Bovine Tuberculosis Episodes

**Figure 1:**
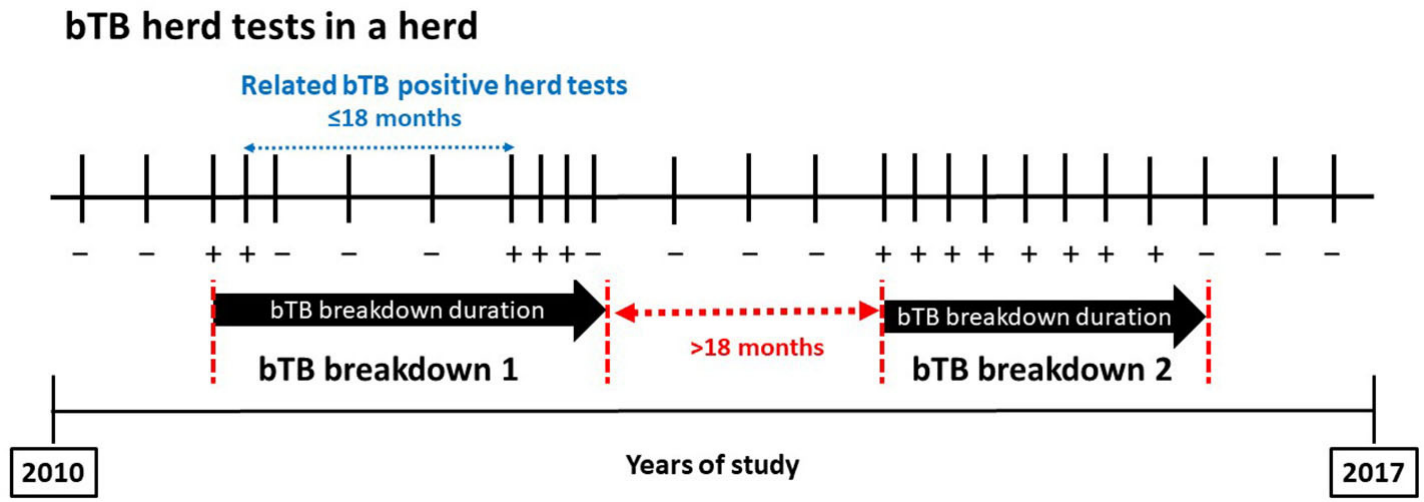
Diagram of bovine tuberculosis (bTB) breakdown definition. Plus and minus signs denote bTB-positive and bTB-negative tests, respectively.

A bTB-positive herd test was defined as a herd test with at least one positive animal in the skin test, IFN-γ assay, or bacteriology. When infection was detected through passive surveillance at the slaughterhouse, this was also considered a bTB-positive event. Two bTB-positive herd tests were considered related if they were separated by <18 months regardless of the number of negative herd tests that could take place in the meantime (Figure 1). Eighteen months was selected as a conservative threshold to increase the power to detect epidemiologically related positive tests since it was close to the median bTB breakdown duration in herds with ≥3 bTB-positive herd tests over the study period (see *Results*). A bTB breakdown was formed by all related bTB-positive herd tests. The bTB breakdown duration was defined as the period elapsed from the first bTB-positive herd test until the first negative herd test following the last related bTB-positive herd test (Figure 1). A bTB breakdown was defined as resolved when the herd did not experience subsequent bTB-positive tests within the next 18 months. For herds experiencing more than one bTB breakdown during the study period, only the longest bTB breakdown was kept in the database. Depopulated herds during a bTB breakdown were excluded to calculate the median bTB breakdown duration.

Available explanatory variables for each bTB breakdown were province of the herd, bTB prevalence in the county the year before the start of the bTB breakdown (extracted from official records provided by the regional veterinary services and available for the period 2011–2017), herd type, median herd size in the year the bTB breakdown started, relative change in herd size prior to the disclosing bTB-positive herd test (percentage difference between the herd size in the first positive herd test of the bTB breakdown and the median herd size the previous year), number of herds sending animals to the bTB breakdown herd (in-degree) and number of incoming animals in the previous 3 years (for breakdowns occurring in 2010–2017), number of days between the disclosing and follow-up tests, and number and proportion of positive animals to the SIT and bacteriology in the disclosing test. For herds in which bTB was disclosed through pre-movement tests or abattoir inspection, the number of animals tested in the previous routine test was used to calculate the relative change in herd size at the disclosing test. Models considering certain risk factors (number of incoming contacts and animals in the 3 years prior to the start of the bTB breakdown and county-level herd prevalence) did not include breakdowns starting before 2010/2011 as no information for those factors was available. Herd size was categorized into three categories based on terciles (small = ≤ 41.1 animals, medium = >41.1– <134 animals, and large = ≥134 animals), relative change in herd size at the disclosing bTB-positive herd test, number and proportion of SIT reactors in the disclosing test, number and proportion of bacteriology-confirmed animals in the disclosing test, in-degree in the 3 years prior to the start of the bTB breakdown, and number of days between the disclosing and follow-up tests were categorized into quartiles, while county-level herd prevalence was categorized into quintiles.

### Survival Analysis

The association between each available predictor variable and the outcome variable “duration of the bTB breakdown” was explored in a survival analysis. The survival distributions for the categories of each of the considered variables were plotted and compared using the log-rank test. These analyses accounted for left truncation (i.e., incomplete information for the start date, for herds with bTB breakdowns already started before January 2010) and right censoring (i.e., incomplete information for the end date, for herds with ongoing bTB breakdowns at the end of the study period, December 2017) as seen elsewhere (24). Here, the included truncation time was days between the bTB disclosure date (in 2007–2009) and the first test in 2010. Depopulated herds were also considered right-censored observations.

Univariable Cox proportional hazards models were fitted to compare the hazard ratios of resolving an outbreak for herds depending on the covariates *Z*_1_…*Z*_*k*_, so that:

$$\begin{array}{l} \lambda \left(t,Z_{i}\right)=\lambda_{0}\left(t\right)e^{Z_{i}\beta_{i}}, \end{array}$$

were *t* represents the survival time, λ_0_(*t*) is the baseline hazard function, and β_*i*_ is the regression coefficient of covariate *Z*_*i*_ (25). Variables considered in the analyses were production type, number of SIT reactors, and number of confirmed animals through bacteriology in the disclosing test, herd size, relative change in herd size, location at a province level, in-degree, number of incoming animals, and county-level herd prevalence. Variables with *p* ≤ 0.2 were then considered in a multivariable Cox proportional hazards model. Categorical variables were preferred over continuous based on Akaike's information criteria (AIC) scores. The choice of variable to include in the model for correlated variables (in-degree and number of incoming animals) was based on the AIC (26). The assumption of proportional hazards was evaluated by computing Schoenfeld residuals for each of the study variables. For variables that did not satisfy the proportional hazards assumption (i.e., the true hazard ratio does not change over time), we introduced time-dependent effects β_i_(t) using a parametric continuous function to construct time-dependent covariates (27, 28).

### Case-Control Study

A case control study to identify risk factors associated with herds with an increased duration of bTB breakdowns (“chronic herds”) was also carried out. For this, we defined a case as any herd with a bTB breakdown duration equal to or greater than 784 days. This 784-day threshold was selected as our aim was to identify bTB breakdowns with an unusually long duration for Castilla y Leon, and this included only the top 25% longer resolved bTB breakdown of herds with ≥3 bTB-positive herd tests (see *Results*). A control was any herd with a bTB breakdown duration less than or equal to the median bTB breakdown duration in the whole study population, i.e., irrespective of the number of positive herd tests (133 days, see *Results*) as previously performed (10), that was resolved without recurring to depopulation. In this case, calculation of the bTB breakdown duration for breakdowns starting in 2007–2009 or finishing in 2018 included the time in those years. For case herds that underwent depopulation during the bTB breakdown, the date of the clearance/end of the bTB breakdown was set to the date of the depopulation. When herds tested positive following depopulation, this was considered a new bTB breakdown.

Each of the potential risk factors listed above was then tested in a univariable logistic regression model using the chronic status (case/control) as the outcome variable. Risk factors that were significant in the univariable model at a liberal *p* < 0.20 were considered for inclusion in a multivariable model. Multicollinearity between potential covariables was assessed using the variance inflation factor (VIF) to ensure a mean VIF of <5 among the variables (29) before being offered to the multivariable model. For correlated variables, AIC was used again to perform variable selection (26). The final model considered the selected risk factors along with significant two-way biologically plausible interactions and was built using a backward selection procedure based on a likelihood ratio test (*p* ≥ 0.05). Results in the model were expressed as odds ratios (ORs) and 95% confidence intervals (CIs). The Hosmer–Lemeshow statistic was used to test the goodness of fit of the model (30).

Statistical analyses were performed using R version 3.5.0 (31). Survival analyses and Cox proportional hazards models were built using the survival (32) and survminer (33) packages, and model fitting assessment was performed through the ResourceSelection (34) package in R.

## Results

According to the definition used in this study, there were 3,550 (20%) bTB-positive herds out of 17,793 tested herds in Castilla y Leon at some point during the period 2010–2017. Beef was the predominant production type among the bTB-positive herds (3,026/3,550, 85.2%), followed by dairy (274/3,550, 7.7%), mixed (beef and dairy) (148/3,550, 4.2%), bullfighting (63/3,550, 1.8%), fattening (38/3,550, 1.1%), and raising heifer herds (1/3,550, <0.1%, excluded from further analyses so that the total number of positive herds considered is 3,549). Throughout the study period, the majority of bTB-positive herds was found in the province of Salamanca (*n* = 1,452/3,550, 40.9%), followed by Avila (*n* = 603/3,550, 17%), Leon (*n* = 348, 9.8%), and Segovia (*n* = 299, 8.4%), whereas the rest of the provinces accounted for the remaining 23.9% (*n* = 848) of the total number of bTB-positive herds in Castilla y Leon (Supplementary Figure 2). Out of these bTB-positive herds, 49.5% (*n* = 1,758) experienced only one bTB-positive herd test, 18.1% (*n* = 642) two, and 32.4% (*n* = 1,149) of the herds were positive in three or more herd tests. Four hundred nine herds tested negative at least once between bTB-positive tests within the same breakdown. The median bTB breakdown duration in herds with ≥3 bTB-positive herd tests after the exclusion of 49 herds that were subjected to whole-herd depopulation during the bTB breakdown was 511 days [interquartile range (IQR) = 284–784].

### Survival Analysis

Ninety-five out of the 3,549 bTB-positive herds were excluded from the survival analyses as they were tested only once during the study period. Up to 321 (9.3%) out of the remaining 3,454 bTB-positive herds detected in the period 2010–2017 did not clear the infection during the study period or were depopulated and were therefore considered censored observations in the analysis. In addition, 247 (7.2% out of 3,454 herds) of the bTB breakdowns had started before 2010 and were therefore left-truncated.

**Figure 2:**
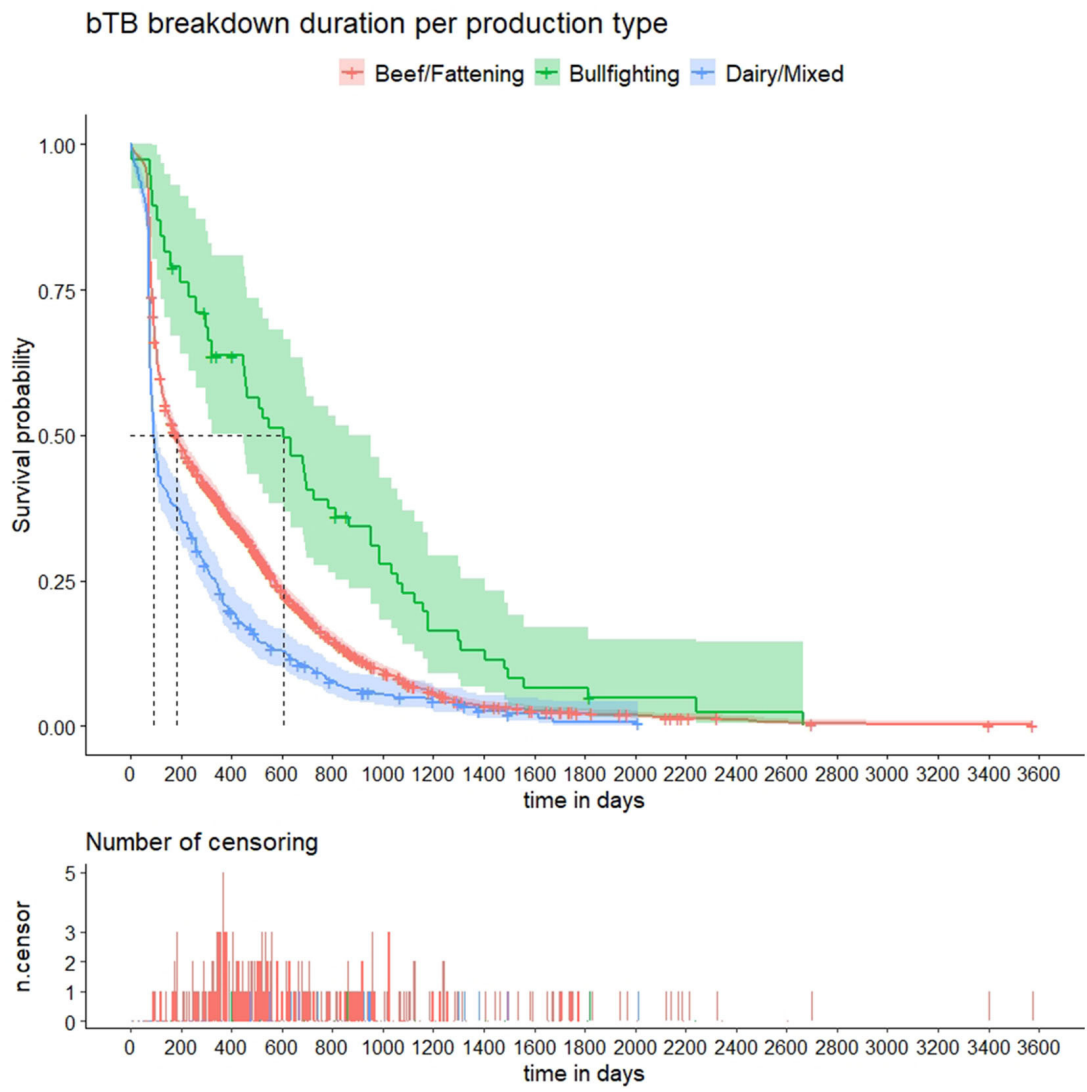
Kaplan–Meier survival estimates of bovine tuberculosis (bTB) breakdown duration by production type. Crosses indicate censored observations. Number of censoring table shows the number of herds that did not clear the infection during the study period or were depopulated during the study period at regular time intervals.

Kaplan–Meier curves for the 10 predictor variables are shown in Figure 2 and Supplementary Figures 3–5. Results of the univariable analyses are shown in Table 1. No data on the number of incoming contacts (and animals) in the 3 years prior to the start of the bTB breakdown and county-level herd prevalence was available for breakdowns starting before 2010 (258 herds) and 2011 (437 herds), respectively (Table 1). Additionally, no information about relative change in herd size was found for 83/3,454 herds, as no routine tests were performed prior to the disclosing test. For the remaining 3,371 herds, relative change in herd size (median = 2.3%, IQR = −7.2–14.3) was not associated with the length of bTB breakdown duration (Table 1 and Supplementary Figure 4). The number of SIT and bacteriology-positive animals in the disclosing test were retained in the models over the proportion of the herd positive to SIT and bacteriology based on better AIC. All the remaining variables (increasing herd size, in-degree, number of incoming animals in the 3 years prior to the start of the breakdown, county-level herd prevalence, number of days between the disclosing and follow-up tests, production type, province, number of SIT reactors and positive to bacteriology animals in the disclosing test) were significantly (*p* <0.001) associated with an increasing time to recover OTF status (Table 1 and Supplementary Figures 3–5). However, the last five variables had non-constant Schoenfeld residuals across time with a large departure of the proportional hazards assumption (Supplementary Figure 6).

**Table 1:** Results from the univariable survival analyses performed on herds with bovine tuberculosis (bTB) breakdowns declared in 2010–2017 in Castilla y Leon.

**Variable (number of herds with information)**	**Levels**	**Number of herds**	**Herds with resolved outbreaks**	**Median time for recovery of OTF status (days) (95% CI)**	**Hazard ratio (95% CI)**	***P*-value**	**Log-rank test**
Relative change in herd size (%) (*n* = 3,371)	1Q (−100 – −7.2)	843	772	166 (146–195)	1 (NA)	–	0.909
	2Q (−7.21–2.3)	842	768	167 (137–205)	0.97 (0.9–1.1)	0.614	
	3Q (2.31–14.3)	855	774	175 (141–221)	0.97 (0.9–1.1)	0.568	
	4Q (14.31–3,800)	831	738	182 (147–218)	0.95 (0.9–1.1)	0.359	
Median herd size in the year the bTB breakdown started (*n* = 3,447)	Small	1,030	963	106 (99–114)	1 (NA)	–	<0.001
	Medium	1,230	1,123	175 (148–205)	0.73 (0.7–0.8)	<0.001	
	Large	1,187	1,040	334 (282–384)	0.54 (0.5–0.6)	<0.001	
In-degree in the 3 years prior to the start of the bTB breakdown (*n* = 3,196)	0	735	694	126 (112–141)	1.29 (1.2–1.4)	<0.001	
	1	744	683	168 (140–203)	1.11 (0.9–1.2)	0.063	
	2–3	915	823	184 (154–219)	1.07 (0.9–1.2)	0.193	
	>3	802	694	217 (182–282)	1 (NA)	–	<0.001
Number of incoming animals in the 3 years prior to the start of the bTB breakdown (*n* = 3,196)	0	735	694	126 (112–141)	1.45 (1.3–1.6)	<0.001	
	1	640	584	141 (126–182)	1.31 (1.2–1.5)	<0.001	
	2–4	964	851	147 (127–181)	1.31 (1.2–1.4)	<0.001	
	>4	857	765	307 (259–356)	1 (NA)	–	<0.001
County-level herd prevalence in the year prior to the start of the bTB breakdown (*n* = 3,017)	0	1,751	1,600	125 (113–139)	1 (NA)		<0.001
	0.01–0.06	571	520	168 (138–233)	0.83 (0.8–0.9)	<0.001	
	0.061–0.1	364	328	210 (169–295)	0.75 (0.7–0.8)	<0.001	
	0.11–0.2	259	222	315 (183–399)	0.63 (0.5–0.7)	<0.001	
	0.21–1	72	59	260 (174–574)	0.51 (0.4–0.7)	<0.001	
Production type (*n* = 3,454)	Beef/fattening	2,978	2,690	183 (168–202)	1 (NA)	–	<0.001
	Bullfighting	60	52	608 (448–865)	0.29 (0.2–0.5)	<0.001	
	Interaction with time				Yes +^a^		
	Dairy/mixed	416	391	92.5 (87–110)	1.83 (1.6–2.1)	<0.001	
	Interaction with time				Yes –^b^		
Province (*n* = 3,454)	Avila	584	532	259 (216–308)	1 (NA)	–	<0.001
	Burgos	270	255	105 (92–120)	1.4 (1.1–1.7)	0.002	
	Interaction with time				Yes +		
	Leon	336	301	98 (91–140)	1.57 (1.3–1.9)	<0.001	
	Interaction with time				Yes –		
	Palencia	127	115	168 (92–224)	1.33 (<1.1–1.8)	0.047	
	Interaction with time				No		
	Salamanca	1,418	1,245	202 (169–251)	0.95 (0.8–1.1)	0.468	
	Interaction with time				Yes +		
	Segovia	290	283	161 (124–181)	1.32 (1.1–1.6)	0.006	
	Interaction with time				No		
	Soria	135	125	188 (126–295)	1.14 (0.9–1.5)	0.323	
	Interaction with time				No		
	Valladolid	59	51	343 (224–524)	0.78 (0.5–1.2)	0.228	
	Interaction with time				No		
	Zamora	235	226	118 (105–153)	1.35 (1.1–1.7)	0.004	
	Interaction with time				No		
Number of SIT reactors in the disclosing test (*n* = 3,454)	0	777	687	484 (441–524)	0.24 (0.2–0.3)	<0.001	
	Interaction with time				Yes +		
	1	1,518	1,401	112 (105–125)	1 (NA)	–	<0.001
	2–4	945	864	146 (126–182)	0.8 (0.7–0.9)	<0.001	
	Interaction with time				No		
	≥5	214	181	296 (233–412)	0.51 (0.4–0.6)	<0.001	
	Interaction with time				Yes +		
Number of positive to bacteriology in the disclosing test (*n* = 3,454)	0	2,174	2,050	98 (97–101)	1 (NA)	–	<0.001
	1	904	785	503 (483–530)	0.19 (<0.2–0.2)	<0.001	
	Interaction with time				Yes +		
	2	164	136	602 (547–707)	0.14 (0.1–0.2)	<0.001	
	Interaction with time				Yes +		
	≥3	212	162	750 (672–853)	0.08 (<0.1–0.1)	<0.001	
	Interaction with time				Yes +		
Number of days between the disclosing and follow-up tests (*n* = 3,454)	0–70	765	666	111 (70–183)	1 (NA)	–	<0.001
	71–90	989	930	84 (84–85)	1.13 (0.9–1.3)	0.083	
	Interaction with time				No		
	91–124	826	763	120 (118–182)	0.64 (0.6–0.7)	<0.001	
	Interaction with time				Yes +		
	>124	874	774	380 (330–458)	0.29 (0.2–0.3)	<0.001	
	Interaction with time				Yes +		

a *+ Interaction with time increase the value of the coefficient*.

b *− Interaction with time decrease the value of the coefficient*.

The multivariable Cox regression model, run on 3,001 herds with complete information, contained all predictor variables except relative change in herd size (%) and in-degree. The final Cox model was fitted with both time-fixed (production type and number of SIT reactors in the disclosing test) and time-varying coefficients (province, number of days between the disclosing and follow-up tests, and number of positive to bacteriology in the disclosing test), and obtained coefficients did not differ substantially (<19% change) compared with those estimated in the univariable models except for production type, province, and number of SIT reactors in the disclosing test (Tables 1, 2). Time to recover OTF status was not significantly shorter in dairy than in beef/fattening (and bullfighting) herds (*p* = 0.555, HR = 1.04, 95% CI 0.9–1.2; Table 2). The probability of resolving the bTB breakdown over time was lower in herds with no reactors in the disclosing test (thus detected by other means such as passive surveillance) and herds with ≥5 reactors compared with those with just one reactor (Table 2). Province also affected significantly the expected herd breakdown duration (Table 2). Time required to recover OTF status was longer with increasing herd size, number of incoming animals (although not linearly), number of days between the disclosing and follow-up tests, and county-level herd prevalence. There was also a trend of increasing hazard of longer bTB breakdowns associated with the number of bacteriology-positive animals in the disclosing test, although differences with the baseline category (no reactors) decreased over time (Table 2).

**Table 2:** Results from the multivariable survival analyses performed on herds with bovine tuberculosis (bTB) breakdowns declared in 2010–2017 in Castilla y Leon.

**Variable (number of herds with information)**	**Levels**	**Hazard ratio (95% CI)**	***P*-value**
Median herd size in the year the bTB breakdown started (*n* = 3,447)	Small	1 (NA)	–
	Medium	0.73 (0.7–0.8)	<0.001
	Large	0.6 (0.5–0.7)	<0.001
Number of incoming animals in the 3 years prior to the start of the bTB breakdown (*n* = 3,196)	0	1.17 (1–1.3)	0.007
	1	1.06 (0.9–1.2)	0.356
	2–4	1.19 (1.1–1.3)	0.001
	>4	1 (NA)	–
County-level herd prevalence in the year prior to the start of the bTB breakdown (*n* = 3,017)	0	1 (NA)	–
	0.01–0.06	0.92 (0.8–1)	0.114
	0.061–0.1	0.86 (0.8–0.9)	0.017
	0.11–0.2	0.74 (0.6–0.9)	<0.001
	0.21–1	0.68 (0.5–0.9)	0.005
Production type (*n* = 3,454)	Beef/fattening	1 (NA)	–
	Bullfighting	1.09 (0.7–1.6)	0.666
	Dairy/mixed	1.04 (0.9–1.2)	0.555
Province (*n* = 3,454)	Avila	1 (NA)	–
	Burgos	0.76 (0.6–0.9)	0.017
	Interaction with time	Yes +^a^	
	Leon	0.93 (0.8–1.1)	0.496
	Interaction with time	No	
	Palencia	1.29 (0.9–1.8)	0.116
	Interaction with time	No	
	Salamanca	0.85 (0.7–1)	0.036
	Interaction with time	Yes +	
	Segovia	0.93 (0.7–1.1)	0.487
	Interaction with time	Yes +	
	Soria	0.56 (0.4–0.7)	<0.001
	Interaction with time	Yes +	
	Valladolid	0.43 (0.3–0.7)	<0.001
	Interaction with time	No	
	Zamora	0.92 (0.7–1.2)	0.515
	Interaction with time	Yes +	
Number of SIT reactors in the disclosing test (*n* = 3,454)	0	0.61 (0.5–0.7)	<0.001
	1	1 (NA)	–
	2–4	0.93 (0.8–1.0)	0.139
	≥5	0.74 (0.6–0.9)	<0.001
Number positive to bacteriology in the disclosing test (*n* = 3,454)	0	1 (NA)	–
	1	0.15 (0.1–0.2)	<0.001
	Interaction with time	Yes +	
	2	0.12 (0.1–0.2)	<0.001
	Interaction with time	Yes +	
	≥3	0.09 (<0.1–0.1)	<0.001
	Interaction with time	Yes +	
Number of days between the disclosing and follow-up tests (*n* = 3,417)	0–70	1 (NA)	–
	71–90	0.75 (0.6–0.9)	<0.001
	Interaction with time	Yes +	
	91–124	0.44 (0.4–0.5)	<0.001
	Interaction with time	Yes +	
	>124	0.25 (0.2–0.3)	<0.001
	Interaction with time	Yes +	

a *+ Interaction with time significantly increased the value of the coefficient*.

*SIT, single intradermal test*.

### Case-Control Study

**Figure 3:**
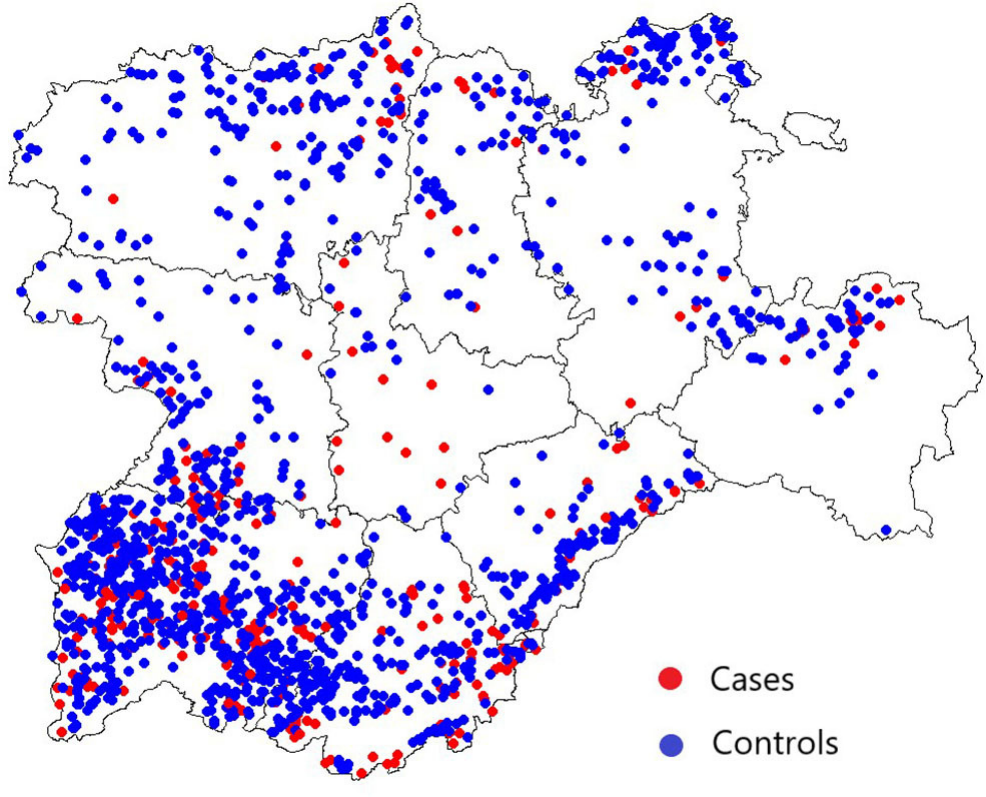
Spatial distribution of case and control herds in Castilla y Leon included in the case-control study. The red and blue circles denote the location of case and control herds, respectively.

A total of 347 herds with a bTB breakdown duration equal to or greater than 784 days (cases) and 1,443 herds suffering a bTB breakdown with a duration ≤ 133 days (controls) were considered in this analysis. No herd had more than one chronic bTB breakdown. Spatial distribution of these chronic and non-chronic herds in Castilla y Leon from 2010 to 2017 is represented in Figure 3. Valladolid was the province with the highest proportion of chronically infected herds among all its herds included in this analysis (11/26, 42.3%), followed by Soria (19/71, 26.8%) (Figure 3 and Table 3). Fourteen case herds underwent depopulation during the bTB breakdown. Forty-seven case herds with ongoing bTB breakdowns at the start of the study period (January 2010) and 30 case herds with ongoing bTB breakdowns after its end (December 2017) were included in the study since the breakdown they experienced already classified them as a case herd.

**Table 3:** Results from univariable and multivariable logistic regression models using the chronic status (case/control) for herds experiencing particularly long breakdowns as the outcome variable.

**Variable (number of herds with information)**	**Exposure level**	**Controls**		**Cases**		**Total**	**OR**	**95% CI**	***P*-value^**a**^**	**OR**	**95% CI**	***P*-value^**a**^**	***P*-value^**b**^**
		**Number**	**%**	**Number**	**%**								
							**Univariable**			**Multivariable**			
Relative change in herd size at the disclosing bTB-positive herd test (%) (*n* = 1,741)	1Q (−100 – −7.2)	339	24.2	89	26.2	428	1	NA	0.891				
	2Q (−7.21–2.3)	359	25.6	84	24.7	443	0.89	0.6–1.2					
	3Q (2.31–14.3)	360	25.7	87	25.6	447	0.92	0.7–1.3					
	4Q (14.31–3,800)	343	24.5	80	23.5	423	0.89	0.6–1.3					
Median herd size in the year the bTB breakdown started (*n* = 1,790)	Small	424	29.4	24	6.9	448	1	NA	<0.001	1	NA	–	<0.001
	Medium	742	51.4	148	42.7	890	3.52	2.3–5.6		4.85	2.6–9.7	<0.001	
	Large	277	19.2	175	50.4	452	11.16	7.2–17.9		9.45	4.8–19.8	<0.001	
In-degree in the 3 years prior to the start of the bTB breakdown (*n* = 1,739)	0	377	26.2	54	18	431	0.57	0.4–0.8					
	1	330	22.9	74	24.7	404	0.89	0.6–1.3					
	2–3	405	28.1	90	30	495	0.89	0.6–1.2					
	>3	327	22.7	82	27.3	409	1	NA	0.018				
Number of incoming animals in the 3 years prior to the start of the bTB breakdown (*n* = 1,739)	0	377	26.2	54	18	431	0.32	0.2–0.4		0.56	0.3–0.9	0.039	
	1	303	21.1	53	17.7	356	0.39	0.3–0.6		0.83	0.5–1.4	0.49	
	2–4	464	32.2	62	20.7	526	0.3	0.2–0.4		0.49	0.3–0.8	0.007	
	>4	295	20.5	131	43.6	426	1	NA	<0.001	1	NA	–	0.027
County-level herd prevalence in the year prior to the start of the bTB breakdown (*n* = 1,660)	0	900	63.6	104	42.3	1004	1	NA	<0.001	1	NA	–	0.004
	0.01–0.06	260	18.4	61	24.8	321	2.03	1.4–2.9		1.89	1.1–3.1	0.012	
	0.061–0.1	138	9.8	36	14.6	174	2.26	1.5–3.4		1.51	0.8–2.8	0.191	
	0.11–0.2	92	6.5	30	12.2	122	2.82	1.8–4.4		1.34	0.7–2.6	0.394	
	0.21–1	24	1.7	15	6.1	39	5.41	2.7–10.5		2.78	1–7.4	0.044	
Production type (*n* = 1,790)	Beef/fattening	1,203	83.4	305	87.9	1508	1	NA	<0.001				
	Bullfighting	6	0.4	19	5.5	25	12.49	5.2–34.5					
	Dairy/mixed	234	16.2	23	6.6	257	0.39	0.3–0.6					
Province (*n* = 1,790)	Avila	208	14.4	70	20.2	278	1	NA	<0.001	1	NA	–	0.002
	Burgos	144	10	10	2.9	154	0.21	0.1–0.4		1.06	0.4–2.6	0.903	
	Leon	178	12.3	24	6.9	202	0.40	0.2–0.6		1.43	0.6–3.2	0.408	
	Palencia	57	4	9	2.6	66	0.47	0.2–0.9		0.55	0.1–1.8	0.358	
	Salamanca	544	37.7	155	44.7	699	0.85	0.6–1.2		1.07	0.6–1.9	0.82	
	Segovia	128	8.9	24	6.9	152	0.56	0.3–0.9		1.55	0.7–3.5	0.285	
	Soria	52	3.6	19	5.5	71	1.09	0.6–1.9		4.73	1.9–11.7	<0.001	
	Valladolid	15	1	11	3.2	26	2.18	0.9–4.9		5.32	1.5–17.4	0.007	
	Zamora	117	8.1	25	7.2	142	0.63	0.4–1		1.91	0.8–4.4	0.135	
Number of SIT reactors in the disclosing test (*n* = 1,790)	0	78	5.4	143	41.2	221	16.93	11.9–24.2		1.69	1.1–3	0.05	
	1	831	57.6	90	25.9	921	1	NA	<0.001	1	NA	–	<0.001
	2–4	461	31.9	83	23.9	544	1.67	1.2–2.3		1.37	0.8–2.2	0.198	
	≥5	73	5.1	31	8.9	104	3.92	2.4–6.3		2.75	1.25.9	0.011	
Number positive to bacteriology in the disclosing test (*n* = 1,790)	0	1,321	91.5	102	29.4	1423	1	NA	<0.001	1	NA	–	<0.001
	1	109	7.6	150	43.2	259	17.82	13–24.6		26.53	16.3–44.1	<0.001	
	2	10	0.7	32	9.2	42	41.44	20.5–91.1		25.48	9.1–75.9	<0.001	
	≥3	3	0.2	63	18.2	66	271.9	98.7–1,126.5		89.96	28.1–408	<0.001	
Number of days between the disclosing and follow-up tests (*n* = 1,790)	0–70	382	26.5	62	17.9	444	1	NA	<0.001	1	NA	–	<0.001
	71–90	601	41.6	66	19	667	0.68	0.5–0.9		0.93	0.5–1.6	0.792	
	91–124	401	27.8	76	21.9	477	1.17	0.8–1.7		1.49	0.9–2.6	0.146	
	>124	59	4.1	143	41.2	202	14.93	10–22.6		14.4	7.6–27.7	<0.001	

a *Walds test*.

b *Chi-square test*.

*bTB, bovine tuberculosis; OR, odds ratio; SIT, single intradermal test*.

The majority (>80%) of both case and control herds were beef/fattening (Table 3). Overall median herd size in the year the bTB breakdown started was 77 (IQR = 41.1–134) and was significantly (*p* < 0.001, Mann–Whitney test) larger for cases (median = 134.8, IQR = 81.3–229) compared to controls (median = 68.3, IQR = 36.4–115).

The proportion of herds with missing information for any given variable was always <8%; out of the 1,790 herds considered in the case-control study, no information for number of incoming contacts (and animals) in the 3 years prior to the start of the bTB breakdown and county-level herd prevalence was available for breakdowns starting before 2010 (51 herds) and 2011 (130 herds), respectively, and were subsequently removed from models considering these variables (Table 3). Moreover, no information about relative change in herd size was found for 49 herds, as no routine tests were performed prior to the disclosing test. In the univariable analysis, nine out of the 10 evaluated variables (all except relative change in herd size at the disclosing bTB-positive test; Table 3) were potentially associated with experiencing a chronic bTB infection. In-degree and number of incoming animals were highly correlated (ρ = 0.74, *p* < 0.001), and the latter was selected for the multivariable model based on better AIC. According to the final multivariable model including 1,657 herds with complete information on all covariates considered, herd size, number of incoming animals in the 3 years before the outbreak, county-level prevalence before the outbreak, the province where the herd was located, number of SIT-positive and bacteriology-confirmed animals in the disclosing test, and an increasing number of days between the disclosing and follow-up tests were all associated with being a chronically infected bTB herd (Table 3), while production type was not. Infected herds with a higher probability of suffering a chronic breakdown were larger (≥134 animals, OR = 9.45 CI = 4.8–19.8), located in Soria and Valladolid (OR = 4.73, CI = 1.9–11.7 and OR = 5.32, CI = 1.5–17.4, respectively), had either no (OR = 1.7, CI = 1.1–3) or above 5 (OR = 2.75, CI = 1.2–5.9) SIT reactors in the disclosing test and at least three (OR = 89.96, CI = 28.1–408) animals confirmed through bacteriology, and were located in counties with a herd prevalence above 0.2% (OR = 2.78, CI = 1–7.4) in the year preceding the start of the bTB breakdown (Table 3). There were no significant interaction terms. The model fitted well the data (Hosmer–Lemeshow test, χ^2^ = 10.29, *p* = 0.245).

## Discussion

The persistence of bTB in certain cattle herds in terms of either herd recurrence or prolonged periods of restriction is a major problem in countries pursuing disease eradication worldwide. Among the factors that may substantially extend the time to recover OTF status and thus hamper eradication programs are the chronic nature of the disease, the presence of wildlife reservoirs, and the lack of performance of diagnostic tools. Several studies have conducted risk factor analyses for chronic bTB herd breakdowns in Europe (10, 35–38), but factors associated with disease persistence in infected herds had not been characterized at this level yet in Spain. Survival and case-control analyses performed in this study revealed the impact of both farm and breakdown characteristics on the probability of disease persistence in infected farms.

In this study, we defined two positive bTB herd tests as potentially related if they were separated by no more than 18 months, the median duration of bTB outbreaks in herds with ≥3 bTB-positive herd tests, which represented 32.4% of the total bTB-positive herds during 2010–2017. Herds with ≤ 2 bTB-positive herd tests were not considered to select the threshold in order to focus on problematic herds. The median bTB breakdown for all bTB-positive herds in Castilla y Leon duration was 133 days, which is in agreement with values reported in Northern Ireland (10, 36).

The univariable survival analyses revealed that production type was associated with longer bTB breakdown durations: unsurprisingly, outbreaks in bullfighting herds were significantly longer compared to beef/fattening and dairy/mixed herds in agreement with previous studies conducted in a different region in Spain (39). Some tests may have differing performances depending on breed type, what could impact the time to recover OTF status (40): in this sense, both the skin test and the IFN-γ assay can have a lower performance in bullfighting cattle due to a combination of stress, difficulties for performing correctly the test due to the temper of the animals, and the lower response to tuberculins/decreased IFN-γ production, what could lead to a higher proportion of false-negative animals remaining in the herd and thus further contribute to the increased outbreak duration (15, 41). In addition, bullfighting herds are extensively managed and therefore can have an increased risk of contact with infected wildlife (42), which could lead to frequent reinfections, thus increasing the length of the outbreak. However, when other variables were considered in the multivariable model, differences were not significant. The absence of a significant effect of the bullfighting production type in outbreak duration may be due to the low sample size of bullfighting herds (<2% of the total herds included in the survival analysis). Interestingly, herd type was identified as a risk factor only in the univariable models (survival analysis and case-control study) but not in the multivariable, thus suggesting that other factors (potentially associated with production type such as province, county-level herd prevalence, and herd size) could be explaining at least part of the apparent effect of this variable in the probability of a herd being classified as a case.

The number of SIT reactors at the disclosing test was significantly associated with both outbreak duration and the odds of a chronic infection: as expected, a higher number of reactors (≥5), suggestive of active circulation of the disease in the herd, was associated with increased duration/risk of being a case herd compared with the reference category (one reactor). However, herds in which no SIT reactors had been found in the disclosing test were also at higher risk (Tables 1, 3) compared to herds with one SIT reactor. These herds (*n* = 777) included herds in which IFN-γ was being used (*n* = 321, 41.3%), what indicates that even in the absence of SIT reactors, there was conclusive evidence of the presence of disease, since the IFN-γ test is only used on units confirmed by bacteriology or based on epidemiological grounds (i.e., multiple herds managed as a single epidemiological unit with one or more having a confirmed bTB infection or that had animals that were in shared pastures with other positive herds) (Supplementary Figure 1). During 2016, both the Bovigam® and the IDvet test versions of the IFN-γ were applied. However, the potential effect of the different versions of the IFN-γ test was not considered in this study, as no information on which version of the IFN-γ assay was used on specific herds was available. In the remaining 456 herds, IFN-γ was not being applied, and infection was therefore found through slaughterhouse surveillance (detection of lesions) or, for OTF herds with a previous history of bTB, bacteriology performed routinely on older animals sent to the abattoir (performed in <1% of the herds of this study). In this case, therefore, the higher duration of breakdowns and increased risk of becoming a chronic herd could be associated with the presence of anergic animals with visible lesions, especially in extensively managed herds, that could be infecting other animals while remaining undetected for some time during the breakdown. When herds in which IFN-γ was applied were removed, the same result was obtained (change in coefficient <9%, data not shown), thus suggesting that herds found through passive surveillance are in fact more prone to become problematic from an eradication standpoint. A median of 5.9% (IQR 4.4–10) breakdowns per year were detected via postmortem analysis (data not shown), although this value is well below the estimates of 27%−46% of all new bTB breakdowns per year reported elsewhere (43, 44), results obtained here highlight the usefulness of abattoir surveillance to complement antemortem tests (and partially overcome limitations in their sensitivity). This limitation in the sensitivity of skin tests could also be related to an increased duration of outbreaks due to the difficulties in the removal of all infected animals in one single herd test and would be partially compensated by the repeated application of the tests (45). Additionally, and to maximize the sensitivity of the diagnostic tests, mandatory training courses for veterinarians conducting SITs organized by the Spanish Ministry of Agriculture and Fisheries, Food and Environment have been in place since 2012 (4).

We also found evidence of time-varying effects for the number of bacteriology-positive animals in the disclosing test, province, and number of days between the disclosing and follow-up tests in the survival analyses, as the mean estimates did not distribute evenly throughout the study time. The interval of time between the disclosing test and the follow-up test was significantly associated with both outbreak duration and the odds of a chronic infection, with herds tested at increased intervals (>124 days; Tables 2, 3) being at higher risk compared with herds subjected to follow-up tests within 2 months after the disclosing test. These results are suggestive of the potential benefits of short retesting intervals, which would allow the early removal of positive animals from the herd to reduce the risk of bTB spread.

In the case-control study, cases were selected among herds experiencing bTB outbreaks in the top 25% of the distribution of durations (>784 days, ~26 months) in order to focus on outlier herds in the Castilla y Leon context. This threshold is well above the 1-year threshold used to differentiate chronic infections arbitrarily selected in previous studies in Ireland (46) and Northern Ireland (10, 37). Other studies conducted in the UK used periods of >240 days (twice the minimum restriction period for a confirmed bTB breakdown) (35) and >6 months (38).

The case-control study further revealed the effect of farm-level management and outbreak characteristics with bTB persistence in infected herds: an increased number of bacteriology-positive animals in the disclosing test were significantly associated with increased odds of experiencing long-duration breakdowns. These findings are consistent with a higher risk of chronic infection in herds with multiple SIT reactors, as these herds are more likely to have a higher proportion of animals confirmed through bacteriology. This result may also be related to a higher bacterial excretion in animals with an advanced disease stage and thus leading to a higher risk of bTB persistence due to the increase of the time of contact with susceptible animals (8).

Even though chronic case herds were widespread throughout Castilla y Leon (Figure 3), infected herds located in specific provinces were subjected to longer breakdown durations and a higher probability of becoming chronically infected (Table 3). Interestingly, the two provinces with the highest indication of an increased risk, Soria and Valladolid, hold a relatively low proportion of the population [2.1% (369/17,793) and 1.9% (331/17,793) of the herds considered in the study period, respectively] and had relatively high herd level prevalences (1.5 and 3.7% in 2016, respectively) (47). To date, there is no substantial evidence of disease spillover from wildlife to cattle in these provinces, and further investigations are needed to clarify the reasons for this increased risk.

The association between an increased herd size and the risk of experiencing a chronic outbreak could be explained with the increased odds of finding reactors (both true and false positive) in larger herds (45, 48), since herd size is a known risk factor for bTB detection (19, 37). According to our study definition of a case herd (experiencing a bTB outbreak with duration ≥784 days), the likelihood of case herds not being truly bTB infected can be considered minimal, and therefore this finding may suggest an increased herd sensitivity of the tests in the eradication program in larger herds, as previously reported in the same region (45). Still, bTB infection was not confirmed through bacteriology in 102/347 herds, some of which could have even recovered the OTF status while experiencing what was defined here as a bTB outbreak. Lack of bacterial isolation in those herds could be related to a very small number of culled animals that would be subjected to postmortem analyses due to a very low level of disease, although the presence of repeated cross-reactions due to non-tuberculous bacteria cannot be ruled out.

The number of incoming animals in the 3 years preceding the start of the bTB breakdown was retained in the case-control logistic model based on AIC and in agreement with findings in the survival analysis, even though no significant differences between the variable categories were observed. Still, several studies have consistently evidenced that cattle movements may be important in bTB transmission (49, 50), and even if positive farms were not clustered in the movement network in the region under study here, there was an association between increased connectivity and positivity at the farm level in a previous study (17).

Both survival and case-control studies showed that a county-level herd prevalence above 0.2% in the year prior to the start of the bTB breakdown was significantly associated with an increased risk of experiencing chronic bTB breakdowns. This result is in agreement with previous studies that identified proximity to infected neighbors as a risk factor for persistent bTB infection in Spain (18) and Ireland (14) or prolonged outbreak duration in Northern Ireland (10, 36). Local sources of infection that could contribute to this effect include local movements (51), contact with infected cattle and wildlife reservoirs (52), or environmental sources of *M. bovis* (53), thus contributing to disease recurrence or local persistence (6, 12, 54).

The inclusion of these variables (incoming animals in the 3 previous years and county-level herd prevalence in the previous year) in the final models forced the elimination of outbreaks starting before 2011 (*n* = 437 outbreaks), since no information on them was available for these outbreaks. These outbreaks had been considered to estimate the overall median duration of outbreaks in the region and the threshold for selection of chronic herds. Nevertheless, if these are excluded for these calculations, no major differences were observed (median duration of outbreaks remains at 113 days, and the threshold used to define chronic herds increases from 784 to 844 days), suggesting that they are not significantly affecting the study definitions.

Other factors not explicitly included in the study that could be related to increased duration of outbreaks include wildlife variables or the presence of concurrent infections compromising the diagnostic sensitivity. The effect of paratuberculosis in bTB diagnostic tests has been described in the past in Spain (5, 55, 56) and elsewhere (57–59). Similarly, cross-reactivity with environmental saprophytic mycobacteria could also artificially increase breakdown duration due to the presence of unspecific SIT reactions (60, 61). However, by selecting herds with ≥3 bTB-positive herd tests in which the disease is typically confirmed through bacteriology (~74% of all bTB breakdowns in herds with ≥3 bTB-positive herd tests were confirmed through bacteriology, and 72% of the 347 case herds) should have minimized the presence of false-positive herds in our study population. Ideally, the epidemiological relatedness between positive herd tests should be demonstrated by characterizing the *M. bovis* strains circulating in the farm using molecular typing techniques [spoligotyping, Variable number of Tandem Repeat (VNTR), and whole-genome sequencing (WGS)], but this information was not available for a large proportion of the herds evaluated here.

Time-varying effect emerges when the proportional hazards assumption is not fulfilled (62), and for this reason, we applied a multivariate Cox model with a mixture of time-varying and time-independent parameters as seen elsewhere (63). This allowed accommodating the time-varying effect observed in two out of the nine variables included in the analyses. In all of them, a decrease in the hazards ratio estimates over time was observed, suggesting that the longer the breakdown lasted, the least important their contribution was. In the survival analyses, left truncation was accounted for 7.2% (out of 3,454) of these herds with already started bTB breakdowns before 2010. However, the fraction truncated was not high enough so that estimates became unstable, as the amount of truncation did not approach or exceed the 50% threshold reported elsewhere (24).

Our findings should be interpreted with caution, as associations found here may not reflect causation. Still, our results demonstrate that certain farm and breakdown characteristics (available at the beginning of the breakdown) can help to predict the probability of experiencing a chronic bTB infection among those already infected, what may help to implement specific measures aimed at preventing reinfection (e.g., increased biosecurity) or decrease residual infection (e.g., early detection and removal to slaughter). Further research is needed to better understand the mechanisms behind persistence of bTB in the region, including the risk factors associated with bTB recurrence and the role of wildlife reservoirs in bTB reinfection and/or maintenance in the herd.

## Data Availability Statement

The data analyzed in this study is subject to the following licenses/restrictions: datasets included in this study are owned by the Official Veterinary Services in Castilla y Leon and include information that cannot be made publicly available. Reasonable data requests can be submitted to the authors of the study and will considered individually. Summary data is provided in the article. Requests to access these datasets should be directed to mingonol@jcyl.es.

## Ethics Statement

Ethical review and approval was not required for the animal study because data from animals used in this study was generated through the normal functioning of the tuberculosis eradication campaigns in Spain and therefore no additional procedures were performed as a result of this study.

## Author Contributions

PP and JA designed the study with the help of AG, JN, and OM, who also provided the data. PP analyzed the data with the help of JA, BR, JB, and JLS in data interpretation. PP and JA wrote the initial manuscript and received substantial feedback from all other authors.

## Conflict of Interest

PP and BR were employed by MAEVA SERVET SL. The remaining authors declare that the research was conducted in the absence of any commercial or financial relationships that could be construed as a potential conflict of interest.
