# Supplementary figures and images for: Evaluation of Risk Factors Associated With Herds With an Increased Duration of Bovine Tuberculosis Breakdowns in Castilla y Leon, Spain (2010–2017)
Source: Front Vet Sci. 2020 Sep 25;7:545328. doi: 10.3389/fvets.2020.545328 (PMC7546324; doi:10.3389/fvets.2020.545328)

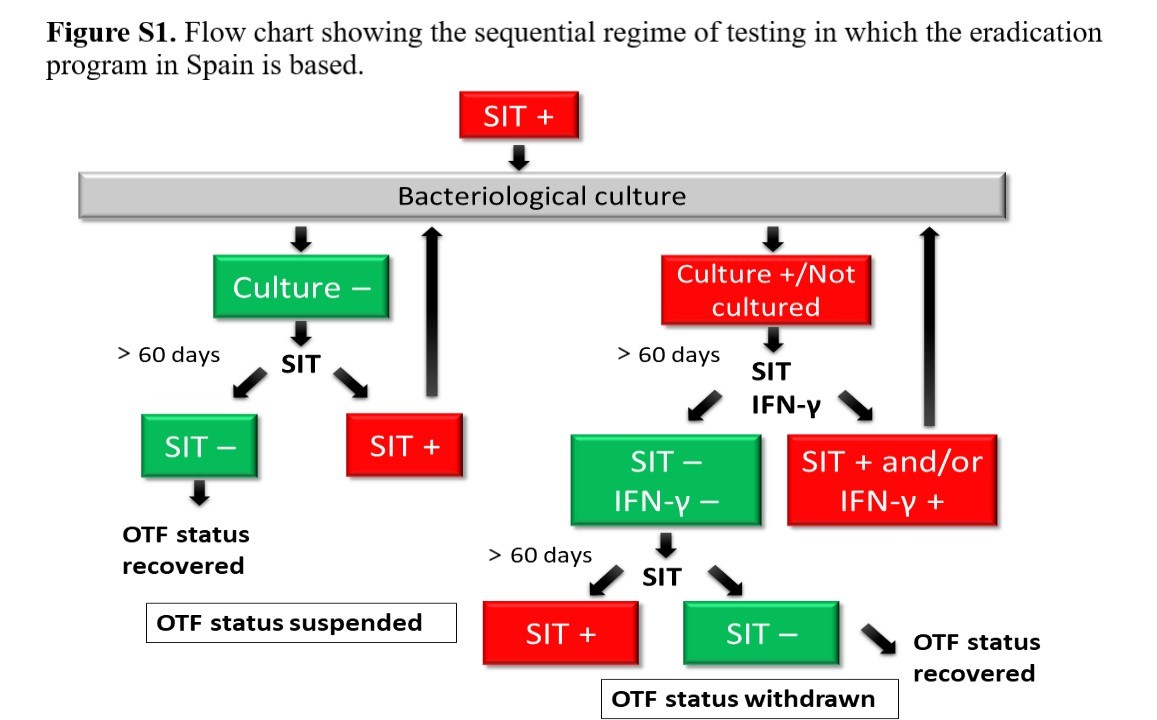

Supplement: Supplementary file 1 [file Image_1.jpg]

Figure S3. Kaplan–Meier survival estimates of bTB breakdown duration

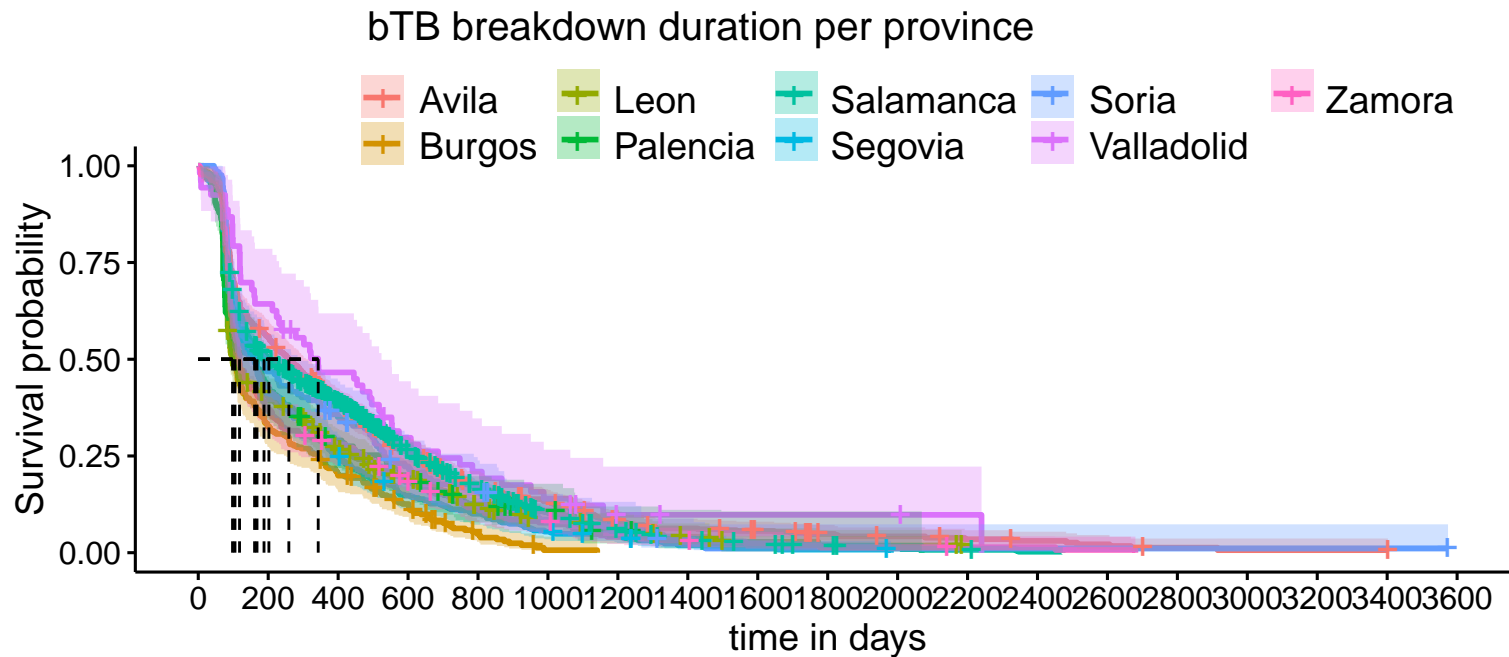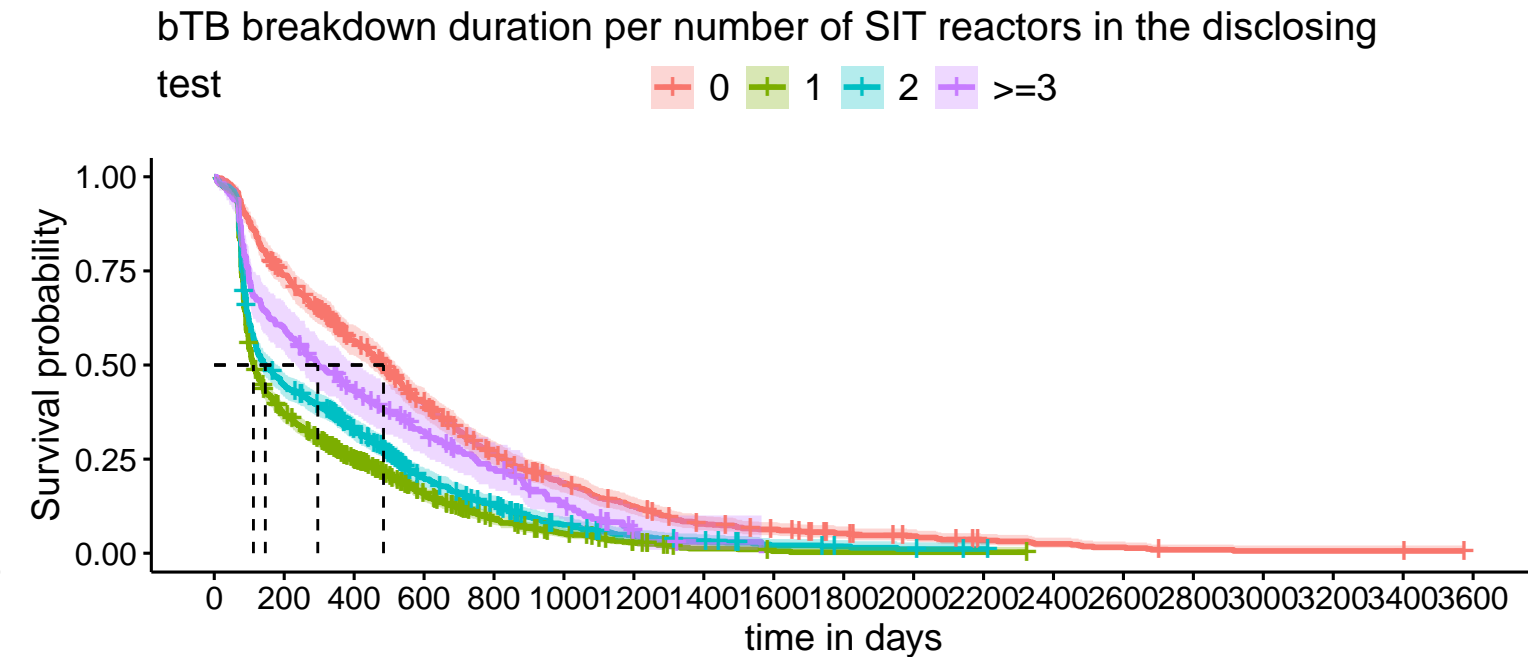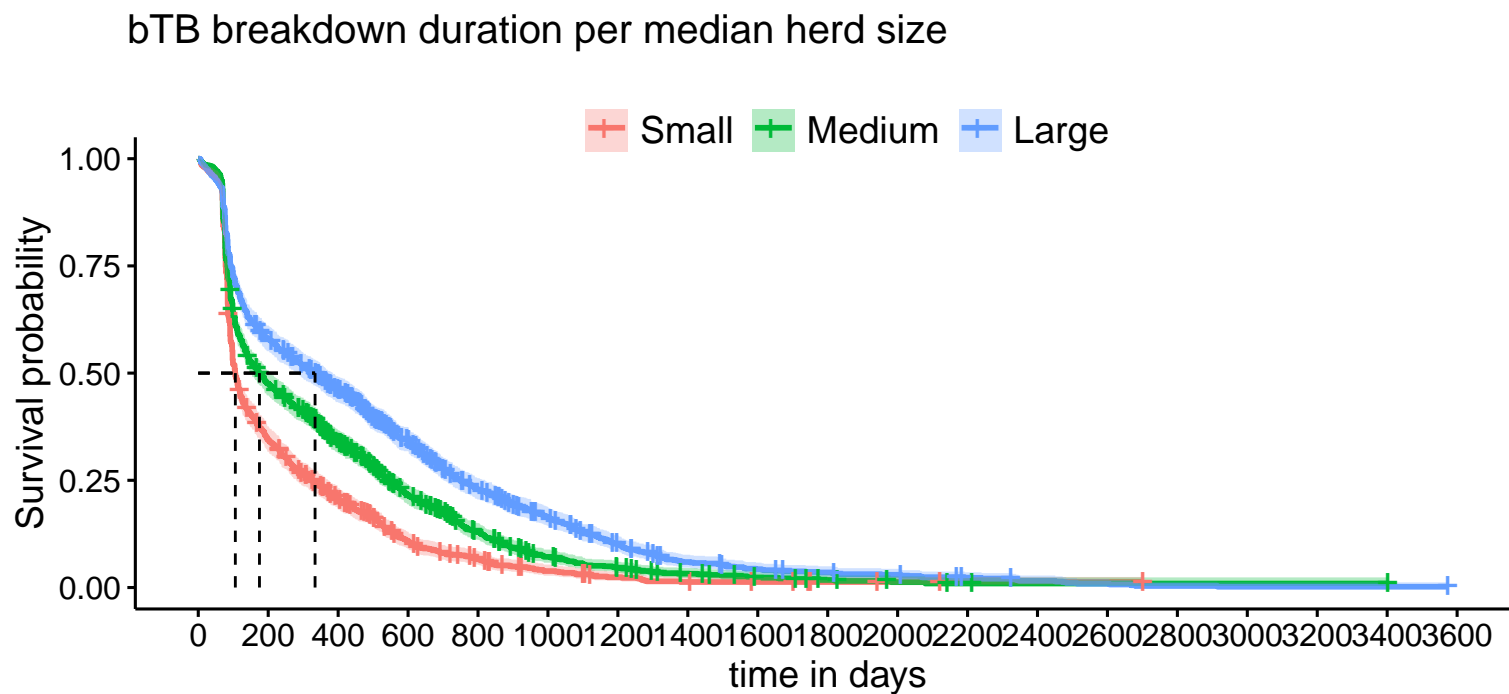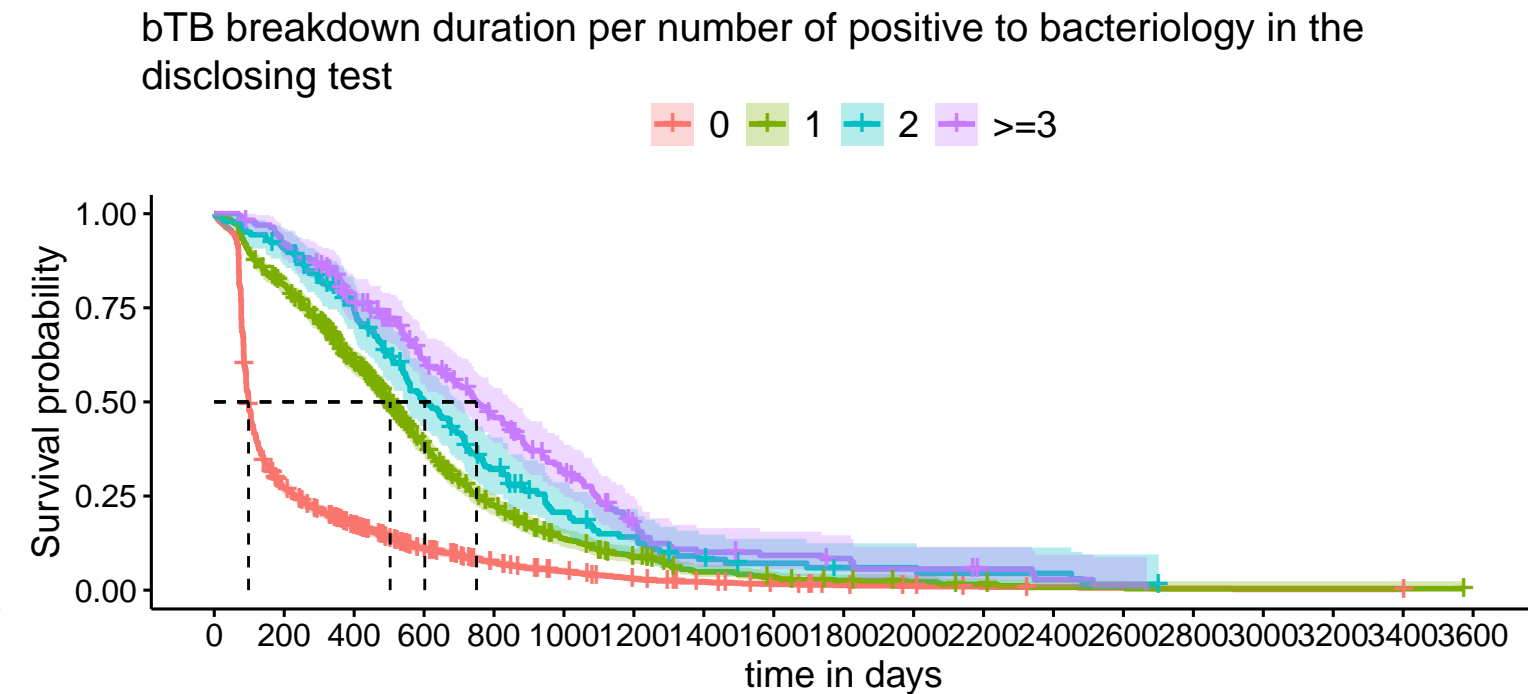

Supplement: Supplementary file 3 [file Image_3.pdf]

**Figure S5.** Kaplan–Meier survival estimates of bTB breakdown duration

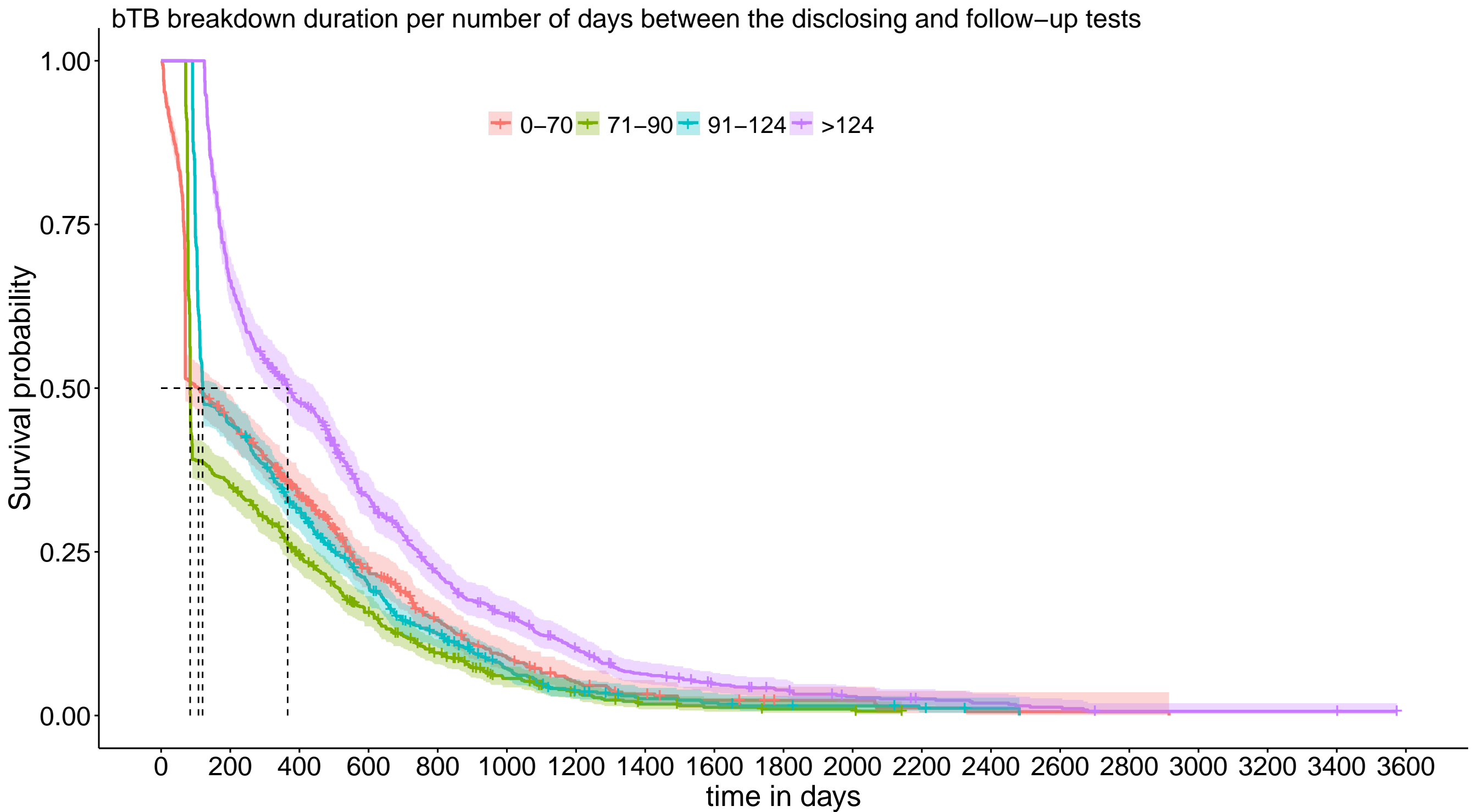

Supplement: Supplementary file 5 [file Image_5.pdf]

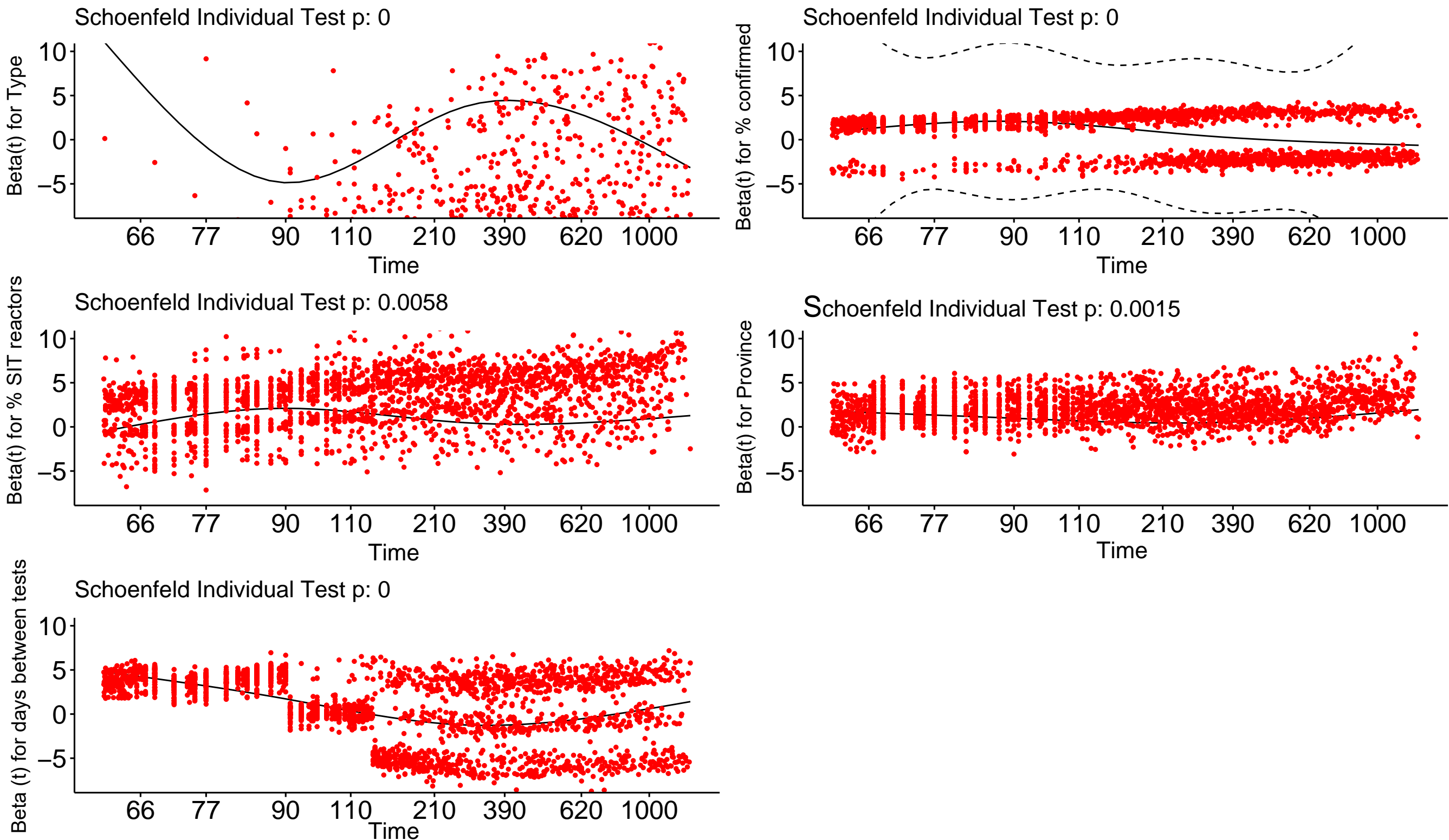

Figure S6. Graphs of the scaled Schoenfeld residuals against the transformed time

Supplement: Supplementary file 6 [file Image_6.pdf]
