# Supplementary material for: Evaluation of Risk Factors Associated With Herds With an Increased Duration of Bovine Tuberculosis Breakdowns in Castilla y Leon, Spain (2010–2017)
Source: Front Vet Sci. 2020 Sep 25;7:545328. doi: 10.3389/fvets.2020.545328 (PMC7546324; doi:10.3389/fvets.2020.545328)
Supplement: Supplementary file 2 [file Image_2.pdf]

**Figure S2**. Total number of herds (A) and number of bTB positive herds (B) per province during 2010-2017 in Castilla y Leon

**A**

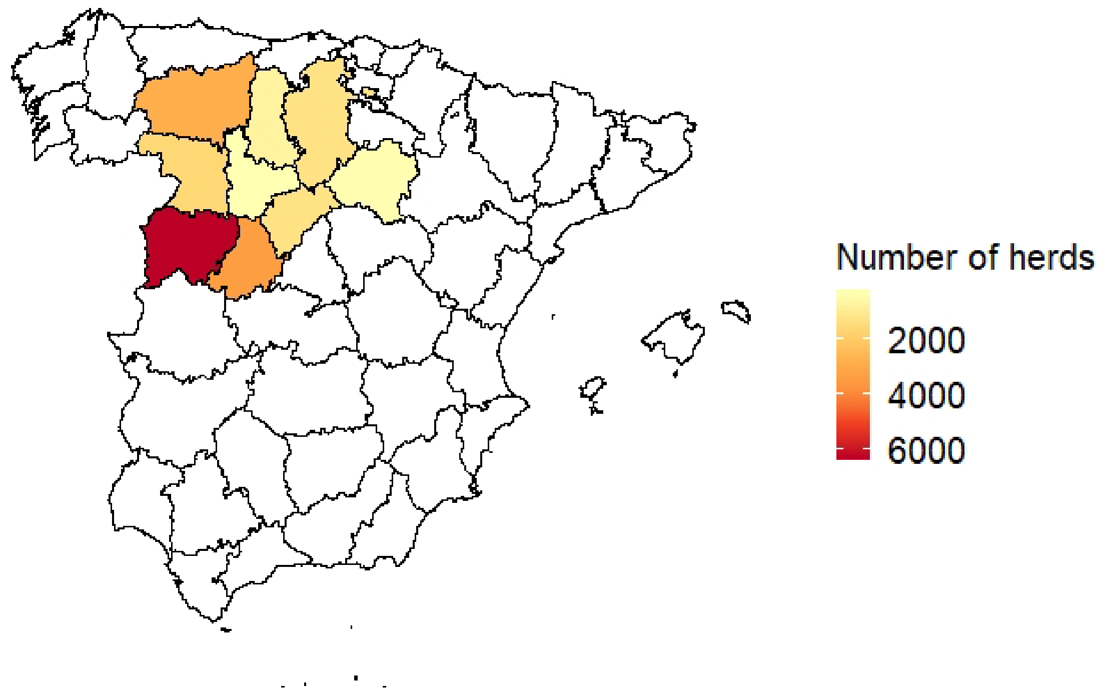


**B**

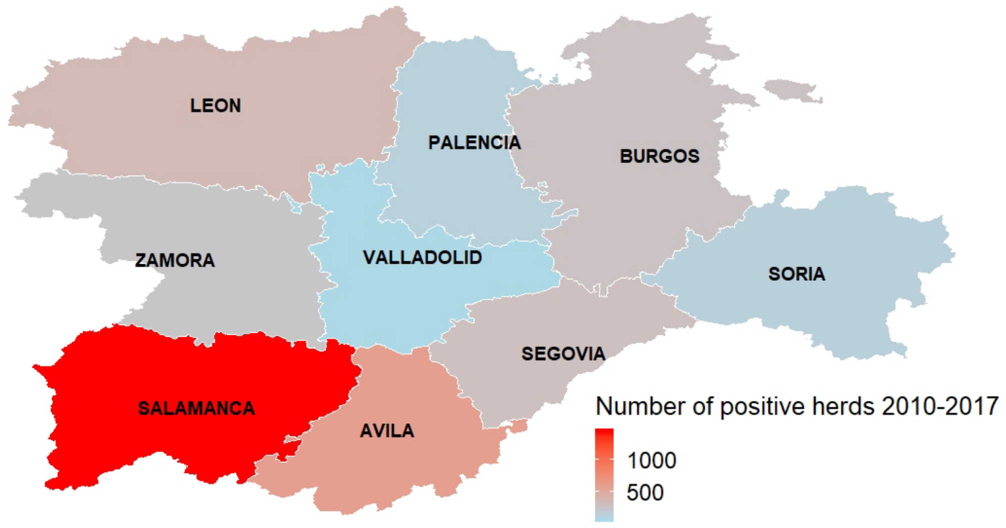


| Province | Number of bTB positive herds (%) |
|---|---|
| Avila | 603 (17) |
| Burgos | 276 (7.8) |
| Leon | 348 (9.8) |
| Palencia | 131 (3.7) |
| Salamanca | 1,452 (40.9) |
| Segovia | 299 (8.4) |
| Soria | 137 (3.9) |
| Valladolid | 62 (1.7) |
| Zamora | 242 (6.8) |
| Total | 3,550 (100) |
