# Supplementary material for: Evaluation of Risk Factors Associated With Herds With an Increased Duration of Bovine Tuberculosis Breakdowns in Castilla y Leon, Spain (2010–2017)
Source: Front Vet Sci. 2020 Sep 25;7:545328. doi: 10.3389/fvets.2020.545328 (PMC7546324; doi:10.3389/fvets.2020.545328)

Figure S4. Kaplan−Meier survival estimates of bTB breakdown duration

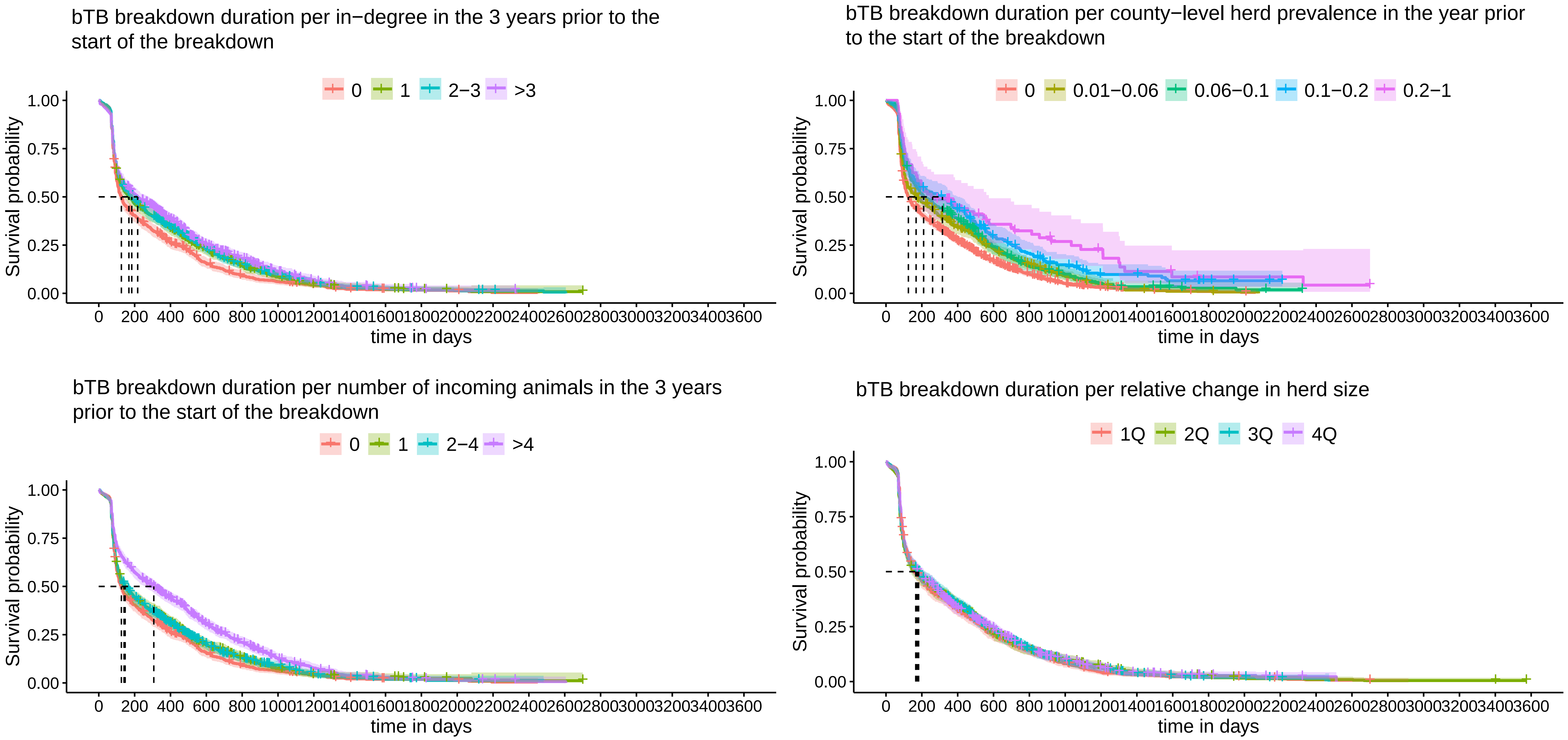

Supplement: Supplementary file 4 [file Image_4.pdf]
